# Low-level laser therapy and exercise for patients with shoulder disorders in physiotherapy practice (a systematic review protocol)

**DOI:** 10.1186/s13643-015-0050-2

**Published:** 2015-04-30

**Authors:** Adedapo W Awotidebe, Gakeemah Inglis-Jassiem, Taryn Young

**Affiliations:** Division of Community Health, Department of Interdisciplinary Health, Faculty of Medicine and Health Sciences, Stellenbosch University, Francie van Zijl Rylaan Drive, Tygerberg Campus, PO Box 19063, Cape Town, 7505 South Africa; Division of Physiotherapy, Department of Interdisciplinary Health Sciences, Faculty of Medicine and Health Sciences, Stellenbosch University, Francie van Zijl Rylaan Drive, Tygerberg Campus, PO Box 19063, Cape Town, 7505 South Africa; Centre for Evidence-based Health Care, Faculty of Medicine and Health Sciences, Stellenbosch University, Francie van Zijl Rylaan Drive, Tygerberg Campus, PO Box 19063, Cape Town, 7505 South Africa

**Keywords:** Low-level laser therapy, Exercise, Physiotherapy, Rehabilitation, Shoulder disorders, Systematic review

## Abstract

**Background:**

Low-level laser therapy is one of the adjunct treatments of choice with exercise therapy for shoulder rehabilitation in physiotherapy clinical practices. Although previous reviews have found little use of low-level laser therapy, there are recent trials whose findings are yet to be systematically reviewed.

**Methods:**

We plan to do a systematic review to assess the effects of low-level laser therapy with exercise and exercise alone in participants who are 18 years and above, with a clinical or radiological diagnosis of various shoulder pathologies. We will search CENTRAL, MEDLINE, CINAHL, PEDro, Science Direct, Scopus and Physiotherapy Choices regardless of publication status. We will hand search for subject-specific journals (PhotoMedicine and Laser Surgery, Lasers in Surgery and Medicine and Journals of Lasers in Medical Science) *and* conference proceedings of World Association for Laser Therapy. Two review authors will independently screen, select studies, extract data and assess the risk of bias based on *a priori* criteria. Disagreements between review authors will be resolved either through discussion or consultation with a third review author. If there are at least two clinically homogeneous studies, we will perform meta-analysis.

**Discussion:**

The findings will shed more light on the benefit of low-level laser therapy as an adjunct treatment to exercise in the management of shoulder disorders. The findings may also inform decision makers in the review and development of guidelines for shoulder rehabilitation in physiotherapy practices.

**Systematic review registration:**

PROSPERO CRD42014013691

**Electronic supplementary material:**

The online version of this article (doi:10.1186/s13643-015-0050-2) contains supplementary material, which is available to authorized users.

## Background

Various shoulder disorders such as sub-acromial impingement, rotator-cuff tear and frozen shoulder are important complaints of shoulder pain and disability and commonest referrals to physiotherapy clinics [1]. Shoulder complaints presenting as painful shoulder, muscular weakness, diminished range of motion and restricted daily activities are reasons for outpatient visits in physiotherapy clinics [1-5]. Physiotherapy is considered the first line of conservative management and inevitably an adjunct treatment for post-surgical interventions [6]. Of all the shoulder complaints treated by the general practitioners (GP) in primary care, between 10% and 30% are referred for physical therapy [1,7].

In a routine physiotherapy clinic, management of various musculoskeletal shoulder disorders is multifaceted and treatment includes a broad spectrum of physiotherapeutic options such as progressive strengthening exercises, strapping, electrotherapy, low-grade joint manipulation/mobilisation therapy, acupuncture, advice and education [7,8]. However, compared to other treatment options, exercise therapy which includes joint and soft-tissue mobilisation, stretching/flexibility, range of motion and gradual strengthening exercises is the mainstay of rehabilitation protocol for shoulder pain [8,9]. In addition to exercise therapy, the use of low-level laser therapy (LLLT) is also evident among physiotherapists in a wide range of musculoskeletal injuries [10-12]. The application of LLLT is non-thermal and is considered safe; however, its use is contraindicated in part or entirely in patients with malignant tumours, epilepsy, light hypersensitivity, applications over thyroid gland, thrombotic deep vein thrombotic area and treatment around developing foetus in pregnant women [12,13]. Similarly, it is contraindicated in patients using steroids because it hinders the effect of laser treatment [14].

The LLLT, also known as phototherapy, is a non-invasive application of non-thermal and low-powered laser light of single wavelength of classes IIIa and IIIb [15]. The protocol for laser treatment in musculoskeletal disorders is associated with better performance in wavelengths of between 632 and 1,064 nm and laser power output of less than 5 mW (class IIIa) or less than 500 mW (class IIIb) [12]. The LLLT is typically administered in a routine therapeutic treatment as either stationary in skin contact or stationary with distance from skin over a maximum of ten trigger or painful points for 90 s at 2,000 Hz [16], 150 s at 3,500 Hz [17], or 5 min at 1,000 Hz [18] per painful point.

Mechanism of action and clinical effect of low-level laser therapy was described by Moshkovska and Mayberry [13] and illustrated in Table 1. All forms of LLLT are used on the site of pathology (for example tendon, joint capsule, cartilage, muscle) or nerve supplying area on acupuncture or trigger points. There was evidence that LLLT *in vitro* studies facilitated the stimulation of fibroblasts and collagen synthesis in connective tissue repair [19,20]. Evidence exists that LLLT, in addition to connective tissue repair, reduces inflammation and modulates pain [10] through reduction of prostaglandin E_2_ (PGE_2_) receptor concentration and inhibition of cyclooxygenase-2 (COX-2) [21,22]. Findings from animal study showed that low-power laser radiation (780 nm) on injured rat sciatic nerve accelerates peripheral nerve regeneration by enhancing axonal growth [23].

**Table 1:** **Mechanism of action of low-level laser therapy** [13]

**Types of effect**	**Mechanisms**	**Outcomes**
Anti-inflammatory effect	Activation of microcirculation	Elimination of oedema
	Prostaglandin level changes	
	Equalisation of osmotic pressure	
Stabilisation of lipid peroxidation	Reactivation of superoxide dysmutase and catalase	Reduction of lipid peroxidation
Analgesic	Activation of neuron metabolism and endorphin level growth	Increase pain threshold
Reparation of wound stimulation	ATP accumulation	Defective epithelisation
	Activation of cellular metabolism	Protein and collagen synthesis
	Increase proliferation of fibroblasts and other cells	Capillary formation
Immune response stimulation	Increase proliferation of immune modifying cells	
	Accelerate maturation of immune modifying cells.	
Reflexogenic effect	Excitation of nerve centres	Stimulation of physiological functions

In recent years, low-level laser therapy is increasingly being combined with exercise than exercise alone for management of pain and functions in various rehabilitation of musculoskeletal injuries [24-26]. In spite of the drive for evidence-based practice in physiotherapy, recommending for widespread use of LLLT, especially in shoulder rehabilitation, is ambivalent because there was insufficient evidence to determine that LLLT contributes to pain relief and increased function [27]. Subsequently, findings from a number of trials on the effectiveness of LLLT on shoulder disorders are not clear-cut. Both positive results [28,29] and no-effect results [30] exist for studies targeting the benefits of LLLT. Although the physiotherapy management of various shoulder complaints is multifaceted, exercise therapy remains the mainstay of treatment [8,31]. For ethical reasons, in studies that evaluated the effect of LLLT, patients also received exercise or other physiotherapeutic treatments in conjunction with laser treatment [16,18,32-34]. It is therefore unclear what specific contribution the LLLT had on shoulder pain, range of motion and physical function. With this perspective, meta-analysis of low-level laser therapy in shoulder disorders over placebo-only treatment would have been appropriate save for the ethical considerations. However, to meet the growing demand of health practitioners and decision makers on evidence regarding LLLT, Green and colleagues reported that evidence is very low on the effectiveness of LLLT on shoulder disorders [27]. The four primary studies reviewed by Green *et al.* [27], England *et al.* [28], Saunders *et al.* [29], Vecchio *et al.* [30], and Tavena [35] were fraught with methodological limitations. Since, additional studies have examined the effectiveness of LLLT on shoulder disorders [16-18,33,36-38]. Recently, one review study [39] examined the effect of LLLT combined with an exercise programme in adults with shoulder pain. The authors did literature appraisal of four of the above studies [16,32,33,37]. This study had several limitations: there was no information on the assessment of risk of bias of the included studies, and this is likely to affect the validity of findings. Moreover, the authors reported a qualitative summary of individual studies to draw conclusions; a meta-analysis of these studies would have provided a more precise estimate of the effects of LLLT on shoulder pain and function. In addition, undertaking subgroup and sensitivity analyses could have impact on the conclusions. It is therefore important and inevitable to conduct systematic reviews and meta-analysis of all these trials.

### Objectives

The aim of this systematic review is to assess the effects of low-level laser therapy with exercise compared to exercise alone in the treatment of shoulder disorders.

The secondary objective is to assess the safety of low-level laser therapy in the treatment of shoulder disorders.

## Methods/Design

### Criteria for considering studies for this review

#### Types of studies

The review will consider randomised controlled trials (RCTs).

#### Types of participants

The review will consider participants who are 18 years and above, with a clinical or radiological diagnosis of various shoulder pathologies by the referring orthopaedic surgeons and physiotherapist and receiving treatments in both in- and outpatient physiotherapy clinics.

#### Types of interventions

The intervention group should have received exercise therapy and either class IIIa (laser power output of less than 5 mW) or IIIb (laser power output of less than 500 mW) LLLT at the site of pathology within the required range of laser treatment protocol for musculoskeletal injuries. The control group should have received exercise therapy and placebo LLLT, and trials that do not have control groups but used one shoulder as treatment and opposite shoulder as a control will be excluded to avoid co-intervention of effect. Participants in the eligible studies would have received LLLT as part of treatment for included shoulder pathologies and information on duration, intensity, dosage, frequency, treatment time and accumulated energy delivered from all sessions must be reported.

#### Types of outcome measures

The primary outcomes will be pain, disability and range of motion related to shoulder injury.

- Pain measured by visual analogue scale (VAS) and other categorical rating scales (the higher the rating, the higher the pain).
- Disability: measures will include the different validated instruments of disability (Shoulder Pain and Disability Index, pain-free muscle strength, 6-minute maximal walking distance, Western Ontario and McMasters Universities Osteoarthritis Index, Roland Morris Disability Index, Oswestry Pain and Disability Index). The mean (standard deviation) disability scores will be used to pool weighted mean differences (if one disability scale is used in all the included studies). Where studies have different scales to measure disability, standardised mean differences (SMDs) will be used.
- Joint range of motion (passive and active).

The secondary outcome measures will include:

- Adverse effect measured by the number of patients experiencing untoward reactions following LLLT treatment.
- Other pain outcomes (for example pain measured at rest, pain on palpation and during physical activities).
- Quality of life measured by Short Form-36 (SF-36).

### Search methods for identification of studies

#### Databases

To identify eligible RCTs for this review, an exhaustive search of the following subject-specific databases will be searched:

- Cochrane Central Register of Controlled Trials (CENTRAL)
- MEDLINE (via EBSCOhost) (1996 to date)
- CINAHL (via EBSCOhost) (1996 to date)
- PEDro (Physiotherapy Evidence Database) (1997 to date)
- Science Direct
- Scopus
- Physiotherapy Choices

There was no known information on when LLLT was introduced as a treatment of choice in physiotherapy practice. However, we commenced the search from 1996 because all the included studies in a previous and related systematic review [27] were published on or before 1995. The search strategy for MEDLINE is detailed in Additional file 1 and will be adapted for other databases.

### Conference proceedings

We will search conference proceedings of World Association for Laser Therapy to identify other relevant studies for inclusion in this review. We will also send emails to all the invited speakers, oral and poster speakers in the last World Association for Laser Therapy 2012 Conference requesting for information on additional unpublished trials relevant to this review.

### Searching other resources

Hand searching of subject-specific journals: *PhotoMedicine and Laser Surgery* (2007-2011), *Lasers in Surgery and Medicine* (1996 to 2014) *and Journals of Lasers in Medical Science* (2010 to present) will be checked manually to identify any other studies. Lastly, we will manually screen a reference list of included studies.

### Data collection and analysis

#### Selection of studies

Two review authors (AD and GIJ) will independently screen and select titles and abstracts of relevant trials. Full texts of the articles will be obtained and assessed for all potential eligible studies. These will be independently assessed to identify studies to be included in this review based on *a priori* inclusion criteria. We will contact the authors of primary studies if further information is needed or some data are missing. We will resolve disagreements on what studies to include in the review through discussion, and should we fail to agree, a third review author (TY) will be contacted to make the final decision on disputed studies.

#### Data extraction and management

The two review authors (AD and GIJ) who will select studies will independently extract the following information from each included trial:

- Characteristics of the studies including source of funding, country, setting, publication date, citation and contact details.
- Participant characteristics including age, sex, socio-demographics, diagnosis criteria, study population.
- Characteristics of LLLT in both intervention and control group, wavelength, class of laser, application procedure, power density, energy dose delivered, treatment time, number of sessions, frequency, average output of laser reported, spot size on the skin, power density and accumulated energy delivered for all sessions.
- Outcome measures for dichotomous parameters, the number of events will be extracted and means and standard deviations for continuous outcomes for estimating the effect of treatment.

Primary authors of the included studies will be contacted if further information is needed and disagreements between review authors will be resolved either through discussion or consultation with a third review author (TY). Data will be captured in the table of included studies.

#### Assessment of risk of bias in included studies

We will assess the risk of bias in included studies independently against the following methodological domains as recommended by the Cochrane Collaboration [40]:

- Sequence generation
- Allocation sequence concealment
- Blinding of patients, health personnel and outcome assessors
- Incomplete outcome data
- Selective outcome reporting
- Other biases

Each of these domains will be judged as: ‘low risk of bias’, ‘high risk of bias’, or ‘uncertain’. We will resolve any disagreement by discussion or arbitration through a third review author.

#### Measurement of treatment effect

For continuous data (for example visual analogue pain rating scale, shoulder disability assessed by validated instruments, shoulder range of motion, and Health-related quality of life), we will calculate mean differences (MD) with corresponding 95% confidence intervals. However, we will use an SMD if different instruments were used to measure the same outcome or construct. Where possible, if shoulder pain were assessed using a binary pain rating scale (that is, pain vs. no pain), and adverse events (number of the events in the treatment group vs. control group), we will calculate risk ratios (RR) with corresponding 95% confidence intervals.

#### Unit of analysis

The unit of analysis will be the patient.

#### Dealing with missing data

We will send an email to the respective trial author (s) to obtain the missing data. We will neither make any assumption nor will we perform imputations.

#### Assessment of heterogeneity

We will assess trials for clinical heterogeneity based on patient characteristics, intervention characteristics, control group and outcome measures. For heterogeneity, visual inspection of the forest plot, Chi square test and I^2^ statistic will be performed. Both Chi^2^ test and I^2^ statistic will be interpreted based on the guidelines recommended by the Cochrane Handbook for Systematic Review of Interventions [41]. The following values are interpreted as thus:

For Chi^2^:

- *p* < 0.05 (strong likelihood of statistical heterogeneity)
- *p* 0.05 < 0.1 (likely to be heterogeneous)
- *p* > 0.1 (not likely to be heterogeneous)

For I^2^ statistic:

- 0%-40% (might not be important)
- 30%-60% (moderately heterogeneous)
- 50%-90% (substantial heterogeneity)
- 75%-100% (considerable heterogeneity)

#### Assessment of reporting bias

We will assess the reporting bias using funnel plots if we have at least ten studies from included studies that are available for meta-analysis. Results of tests of visual inspection of the funnel plots will be interpreted on the basis of other sources of asymmetry (that is heterogeneity).

#### Assessment of the quality of a body of evidence

We will use GRADE approach [42] to evaluate the quality of evidence for individual outcome reported in this review against the following factors that may either increase or decrease the quality of a body of evidence:

- Limitations in the design and implementation
- Indirectness of evidence
- Unexplained heterogeneity or inconsistency of results
- Imprecision of results
- High probability of publication bias
- Presence of dose-response

Each individual outcome will be judged ‘High’, ‘Moderate’, ‘Low’ and ‘Very low’ levels of quality of a body evidence.

#### Data synthesis

The diagnostics of shoulder problem is often problematic and clinical heterogeneity can be expected because of variations in a population’s demographic characteristics and applications of interventions between studies (for example content, frequency, intensity). Therefore, we would perform meta-analysis using random effects model for estimates of intervention effects.

#### Assessment of safety of low-level laser therapy

We will describe the report of adverse effects or complications arising from the use of LLLT in included studies.

#### Subgroup analysis and investigation of heterogeneity

If there are substantial studies available for meta-analysis, we will perform subgroup analyses using RevMan to compare effect estimates to investigate whether LLLT works differently in subset of participants (young participants vs. old participants: 18-35 years vs. >35 years), duration of intervention (short term: <3 months vs. intermediate: 3-6 months vs. long term: >6 months) and subsets of diagnosis (for example types of shoulder disorder).

#### Sensitivity analysis

We will a perform sensitivity analysis to examine how methodological criteria (allocation concealment and blinding) affect the overall treatment effect. Specifically, we will exclude studies that report high/unclear risk of bias and see how it affects the overall treatment effect.

## Discussion

To the best of the authors’ knowledge, there are no published systematic reviews or meta-analysis that has specifically compared the effectiveness of low-laser therapy with and without exercise of the following trials on shoulder disorders (for example, an update from included studies from 1996 onwards). Although the previous systematic review found low evidence for the benefit of low-laser therapy in shoulder rehabilitation [27], we hope the review of recent trials will shed more light on the benefit of low-laser therapy as an adjunct treatment to exercise in the management of shoulder disorders. The findings may also inform decision makers in the review and development of guidelines for shoulder rehabilitation in physiotherapy practices.

## Additional file

Additional file 1:
**MEDLINE search strategy using PubMed.**

## Abbreviations

LLLT: low-level laser therapy
MD: mean difference
OR: odds ratios
RCT: randomised controlled trial
RR: risk ratios
SMD: standardised mean difference
SPADI: shoulder pain and disability index
VAS: visual analogue scale
