# Supplementary material for: Low-level laser therapy and exercise for patients with shoulder disorders in physiotherapy practice (a systematic review protocol)
Source: Syst Rev. 2015 Apr 30;4:60. doi: 10.1186/s13643-015-0050-2 (PMC4423144; doi:10.1186/s13643-015-0050-2)
Supplement: Additional file 1: — MEDLINE search strategy using PubMed. [file 13643_2015_50_MOESM1_ESM.doc]

**Additional file**

Additional file 1: Medline search strategy using PubMed

**SEARCH STRATEGY**

Search history **Results**

1. "arthralgia"[MeSH Terms] **7945**
2. Arthralgia[Text Word] **8832**
3. "shoulder pain"[MeSH Terms] **2930**
4. Shoulder pain[Text Word] **5744**
5. "shoulder impingement syndrome"[MeSH Terms] **1270**
6. Shoulder impingement syndrome[Text Word] **1371**
7. "bursitis"[MeSH Terms] **3911**
8. Bursitis[Text Word] **3607**
9. "rotator cuff"[MeSH Terms] **5061**
10. Rotator cuff[Text Word] **7967**
11. Subacromial impingement syndrome[Text word] **1765**
12. Frozen shoulder[Text Word] **658**
13. Adhesive capsulitis[Text Word] **555**
14. #1 OR #2 OR #3 OR #4 OR #5 OR #6 OR #7 OR #8 OR #9 OR #10 OR #11 OR #12 OR #13 **26526**
15. "laser therapy, low-level"[MeSH Terms] **3189**
16. low level laser therapy[Text Word] **3625**
17. "lasers"[MeSH Terms] **40315**
18. lasers[Text Word] **51989**
19. "laser therapy"[MeSH Terms] **49506**
20. laser therapy[Text Word] **37721**
21. "phototherapy"[MeSH Terms] **28219**
22. Phototherapy[Text Word] **8931**
23. Laser phototherapy[Text Word] **179**
24. LLLT[Text Word] **915**
25. #15 OR #16 OR #17 OR #18 OR #19 OR #20 OR #21 OR #22 OR #23 OR #24 **114681**
26. "rehabilitation"[MeSH Terms] **150416**
27. Rehabilitation[Text Word] **238051**
28. "physical therapy modalities"[MeSH Terms] **125708**
29. Physical therapy modalities[Text Word] **28287**
30. "exercise therapy"[MeSH Terms] **30701**
31. Exercise therapy[Text Word] **27038**
32. "muscle stretching exercises"[MeSH Terms] **953**
33. Muscle stretching exercises[Text Word] **974**
34. "exercise"[MeSH Terms] **120410**
35. Exercise[Text Word] **241777**
36. "musculoskeletal manipulations"[MeSH Terms] **12311**
37. Musculoskeletal manipulations[Text Word] **971**
38. Physiotherapy[Text Word] **12318**
39. #26 OR #27 OR #28 OR #29 OR #30 OR #31 OR #32 OR #33 OR #34 OR #35 OR #36 OR #37 OR #38 **639617**
40. "randomized controlled trials "[MeSH Terms] OR "controlled clinical trials "[MeSH Terms] OR "clinical trials "[MeSH Terms] OR ("random allocation"[MeSH Terms] OR ("double-blind method"[MeSH Terms] OR ("single-blind method"[MeSH Terms] **1086733**
41. "adult"[MeSH Terms] **5578744**
42. adult[Text Word] **4371427**
43. #41 OR #42 **5918156**
44. #14 AND #25 AND #39 AND #40 AND #43 **15**
